# Potential Suitable Habitat Prediction and Distribution Patterns of *Primula* L. in China Under Climate Change

**DOI:** 10.3390/plants15131942

**Published:** 2026-06-24

**Authors:** Lang Huang, Weihao Yao, Chengran Guo, Rui Chen, Bingda Wang, Qingtao Wang

**Affiliations:** 1 Guizhou Academy of Forestry, Guiyang 550005, China; huang_lng@163.com (L.H.); ywh19831027397@163.com (W.Y.); chenruiqiu@yeah.net (R.C.); 2 Key Laboratory of National Forestry and Grassland Administration on Biodiversity Conservation in Karst Mountainous Areas of Southwestern China, Guizhou Academy of Forestry, Guiyang 550005, China; 3 Observation Station of Subalpine Ecology Systems in the Middle Qilian Mountains, Zhangye 734000, China; 15530816503@163.com; 4 College of Landscape and Ecological Engineering, Hebei University of Engineering, Handan 056000, China; 5 Jilin Province Intellectual Property Protection Center, Changchun 130012, China; 18643870003@163.com; 6 College of Life Sciences, Jilin University, Changchun 130012, China

**Keywords:** *Primula*, geographical distribution pattern, MaxEnt model, climate change, suitable area

## Abstract

Climate change is increasingly reshaping species habitat suitability worldwide. *Primula* L., the largest genus in Primulaceae, comprises 404 species in China (including 296 endemic species) and is characterized by high endemism and numerous rare and endangered taxa. However, global warming has intensified habitat fragmentation and loss, while its distribution patterns and key environmental drivers remain insufficiently understood. We compiled 7647 occurrence records of 404 wild *Primula* species in China and integrated 60 environmental variables (climatic, topographic, and soil factors). Using the MaxEnt model combined with ArcGIS spatial analysis, we assessed current and future habitat suitability, identified dominant environmental drivers, and quantified conservation gaps under multiple climate scenarios. Species richness is highly concentrated in Sichuan (186 species), Yunnan (177 species), and Xizang (165 species), with the Hengduan Mountains and eastern Himalayas representing the core distribution area and showing clear peripheral differentiation. The optimized MaxEnt model performed well (AUC = 0.858), identifying temperature seasonality (bio4, 39.8%) and elevation (27.1%) as the main limiting factors. The total suitable habitat area is 268.52 × 10^4^ km^2^, with high-suitability areas mainly distributed in the Hengduan Mountains, southeastern Qinghai–Xizang Plateau, and the Central Mountain Range of Taiwan. Under three shared socioeconomic pathway (SSP) scenarios (SSP126, SSP245, and SSP585), suitable habitat shows a persistent decline, most pronounced under SSP585 in the 2090s (−20.73%), accompanied by a 25.86% reduction in low-suitability areas. Localized expansion of high-suitability habitats suggests that the Hengduan Mountains and southeastern Qinghai–Xizang Plateau may act as potential climatic refugia. Habitat loss consistently exceeds habitat gain, while the distribution centroid shifts westward and northwestward, with migration distances increasing under higher-emission scenarios. Conservation gap analysis indicates that 90.01% of high-suitability habitats lie outside the current protected area network, revealing a strong mismatch between biodiversity hotspots and conservation coverage. These findings highlight the urgent need to expand protected areas and establish micro-reserves in key gap regions (southwestern Sichuan, northwestern Yunnan, southeastern Xizang, and southern Gansu), and to integrate climate-driven migration corridors into conservation planning to support long-term alpine plant persistence under climate change.

## 1. Introduction

Climate change constitutes a profound and irreversible global environmental crisis, driving widespread range shifts in species and fundamentally reshaping community composition and ecosystem functioning in terrestrial biomes [1]. The vulnerability of species to these changes is critically dependent on both the magnitude of shifts in temperature and precipitation and their intrinsic genetic structure, ecological plasticity, and specific environmental tolerances [2]. Climatic variables, as primary determinants of species distributions and macro-scale vegetation dynamics, dictate the expansion, contraction, or relocation of potential habitats through their spatiotemporal heterogeneity [3]. Consequently, unraveling the mechanisms underlying biotic responses to climate change and elucidating the resultant alterations in plant distribution patterns have emerged as pivotal research priorities in biogeography and macroecology [4]. Anthropogenic greenhouse gas emissions have persistently intensified global warming since the pre-industrial era. The Sixth Assessment Report of the IPCC projects that the global mean surface temperature will exceed 1.5 °C above pre-industrial levels by the end of the 21st century [5], with even more pronounced warming in high-latitude regions; for instance, winter temperatures in the Arctic are forecast to rise by 6 °C or more by 2100 under the intermediate RCP 4.5 scenario [6]. Empirical modeling further indicates that by 2100, the climatically suitable ranges of numerous taxa could disappear entirely. The distribution of *Castanea sativa* in Turkey, for example, is anticipated to undergo severe contraction or complete loss during this period [7]. These observations highlight that the threats of climate change to species persistence are globally pervasive, although their magnitudes exhibit discernible regional disparities. Given its extensive latitudinal span and climatic diversity, China is projected to experience warming rates that may exceed the global mean [8]. Under such a scenario, the suitable habitats of various endemic and sensitive species are likely to diminish significantly. For instance, the suitable habitat area of *Rhododendron huadingense*, a wild plant under second-class national protection in China, is projected to decline by 99.64% by the 2050s under the high-emission SSP585 scenario, leaving only 7.6 km^2^ [9]. Therefore, conducting research on plant distribution prediction under climate change can provide a scientific basis for the planning of priority conservation areas, the formulation of biodiversity adaptation strategies, and the sustainable management of ecosystems.

Species distribution models (SDMs) are widely used quantitative frameworks for assessing and predicting the impacts of climate change on species’ geographic distributions [10]. By integrating species occurrence records with environmental predictors, SDMs estimate current and future habitat suitability across spatial and temporal scales, thereby providing a robust basis for biodiversity assessment and conservation planning [11]. With advances in geographic information systems (GISs), computational algorithms, and the increasing availability of global biodiversity and environmental datasets, SDMs have become fundamental tools in ecology, biogeography, and conservation science, with broad applications in habitat suitability mapping, conservation prioritization, and invasion risk assessment [12,13,14,15]. Methodologically, SDMs have evolved from early statistical approaches, such as generalized linear models (GLMs) and generalized additive models (GAMs), to more flexible machine learning techniques, including random forests (RFs) and artificial neural networks (ANNs), as well as niche-based models such as ecological niche factor analysis (ENFA), genetic algorithm for rule-set prediction (GARP), and maximum entropy modeling (MaxEnt) [16]. Among these approaches, MaxEnt has gained particular prominence due to its ability to operate effectively with presence-only data, its relatively low sample-size requirements, and its strong predictive performance under data-limited conditions, making it one of the most widely applied SDMs in contemporary ecological research [17]. Recent studies have demonstrated the broad applicability of MaxEnt in predicting species responses to climate change. For example, it has been used to project suitable habitats of medicinal and endemic plants, revealing shifts in distribution driven primarily by climatic variables such as temperature extremes and precipitation seasonality. Specifically, projections indicate that some species may experience habitat expansion under moderate climate scenarios, while others show significant habitat contraction or fragmentation under high-emission pathways, highlighting the combined effects of climate change, topographic heterogeneity, and population dynamics on future distribution patterns [18,19,20].

*Primula* L. is one of the largest genera in Primulaceae, comprising approximately 549 species mainly in temperate and alpine regions of the Northern Hemisphere [21,22,23]. Most species of *Primula* are native to China [24]. In China, 399 species (including 25 varieties and 24 subspecies) have been recorded [25], with additional species (*P. yanbianensis*, *P. jiangyouensis*, *P. xinjingensis*) raising the total to 404 [26,27,28,29]. The genus is widely distributed in China but has high endemism (296 endemic species), with Southwest China as the main diversity center [30,31]. Seven species (e.g., *P. davidii*) are Critically Endangered and ten (e.g., *P. ambita*) are Endangered [32]. Most *Primula* are perennial herbs with showy flowers of high ornamental value [33,34]; *P. malacoides* and *P. vulgaris* are widely cultivated for early flowering [35]. Cultivated since the Ming–Qing dynasties, *Primula* is regarded, together with *Rhododendron* and *Gentiana*, as one of “China’s three major alpine ornamental genera”, valued for its compact habit, vivid colors, and synchronized flowering. As an alpine lineage, it helps maintain high-altitude biodiversity and has medicinal value, being cool in nature, and bitter and pungent in taste, with effects including clearing heat, drying dampness, purging liver and gallbladder fire, and stopping bleeding [36]. Infrageneric classification is controversial. Early systems had limited acceptance and unclear boundaries [37,38]. Later treatments proposed schemes from subgenera to sections, reflecting disagreements over morphology and rank [21,22,39,40,41]. No major infrageneric changes have since occurred, but molecular phylogenetics (ITS, cpDNA) has clarified relationships among sections [24,42,43,44]. This study adopts a widely accepted classification based on morphology, cytology, and molecular data [22]. Current research on *Primula* focuses on phytochemistry, pharmacology, genomics, and taxonomy [45,46,47]; climate-change distribution studies are very limited (only *P. filchnerae* and two sections) [35,48,49]. Owing to climate change, human disturbance, and the sensitivity of herbs [50], wild *Primula* populations are declining, and more species are becoming threatened [51,52]. Thus, their ecological responses to climate change remain poorly understood, and studies on potential distribution and key drivers are lacking, underscoring an urgent need for further research.

To address these knowledge gaps, the present study seeks to elucidate the species–environment relationships of *Primula* in China under current and future climate scenarios. Using the Maximum Entropy (MaxEnt) model and ArcGIS software, together with topographic, climatic, and edaphic environmental data, we aim to: (1) characterize the geographical distribution pattern of *Primula* in China; (2) identify the key environmental variables limiting its distribution; and (3) project the dynamic changes in potential suitable habitats and range centroid shifts under current climatic conditions and future climate change scenarios (SSP126, SSP245 and SSP585). The outcomes of this study will provide direct scientific evidence for conservation planning, germplasm resource management, and ex situ conservation strategies of *Primula* under future climate change.

## 2. Results

### 2.1. Geographic Distribution Pattern of Primula in China

#### 2.1.1. Provincial Scale

**Figure 1:**
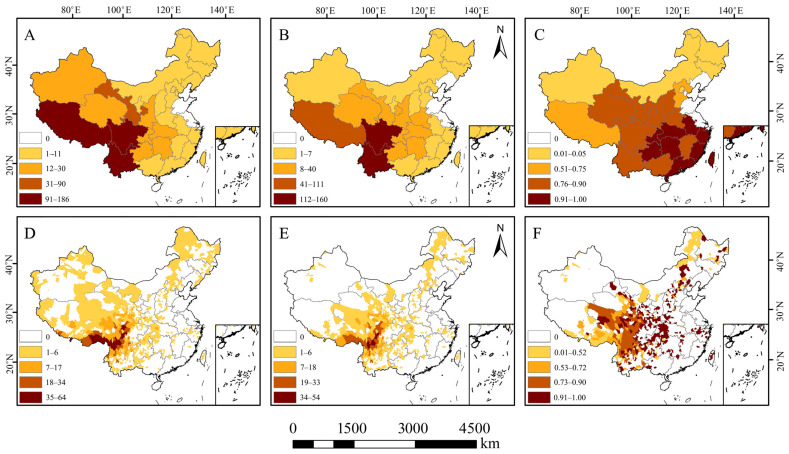
Provincial-level distribution of *Primula*. (**A**) Species richness. (**B**) Endemic species richness. (**C**) Ratio of endemic species to total species. County-level distribution of *Primula*. (**D**) Species richness. (**E**) Endemic species richness. (**F**) Ratio of endemic species to total species.

Wild *Primula* occurred in 28 of China’s 34 provincial regions, comprising 404 taxa (including varieties and forms) (Figure 1A). Based on species richness, five classes were identified. Class I (≥91 spp.) comprised Sichuan, Yunnan, and Xizang; Sichuan had the highest richness (186 spp., 46.04%), followed by Yunnan (177) and Xizang (165). Class II (31–90 spp.) contained only Gansu (48 spp.). Class III (12–30 spp.) included seven provinces (e.g., Guizhou, Chongqing, Shaanxi). Class IV (1–11 spp.) comprised 17 provinces (e.g., Henan, Inner Mongolia, Hebei), where species were disjunct distributed. Class V included six regions (Shanghai, Hong Kong, Macao) with no wild records. Endemism analysis (Figure 1B) showed Sichuan and Yunnan had the most endemics (160 and 139, respectively), followed by Xizang (87). No endemics occurred in Liaoning, Jiangsu, or Hainan. The spatial pattern of endemism differed from that of richness (Figure 1C). Five provinces (Anhui, Fujian, Guangdong, Taiwan, Zhejiang) had 100% endemism (all species endemic). Intermediate rates occurred in Hubei, Chongqing, Guizhou, and Hunan. Relatively high proportions were also found in Henan, Shaanxi, and Qinghai. Low rates were recorded in Hebei and Xizang, and the lowest in Jilin, Beijing, Inner Mongolia, Heilongjiang, and Xinjiang.

#### 2.1.2. County Scale

**Figure 2:**
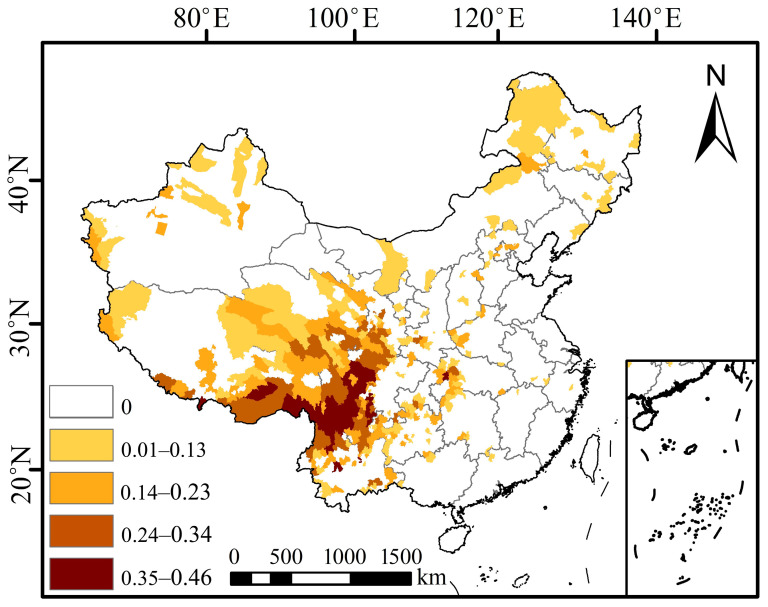
County-level distribution map of species density of *Primula*.

Wild *Primula* exhibited a highly heterogeneous distribution across 648 county-level administrative regions in China, showing a core–periphery pattern centered in the Hengduan Mountains of southwestern China. Fourteen counties recorded >35 species, including Shangri-La City, Muli Tibetan Autonomous County, Kangding City, Deqin County, and Zayü County, all located at the junction of Sichuan, Yunnan, and Xizang. Species richness was highest in Shangri-La City (65 species), followed by Muli County (63), Kangding City (62), Deqin and Zayü (48 each), Milin (43), and Motuo (41), highlighting the southern Hengduan Mountains and eastern Himalayas as the core distribution area of *Primula* in China (Figure 1D). A total of 20 counties recorded ≥30 species, whereas 354 counties contained only 1–2 species, indicating a strongly uneven distribution. Endemic species were recorded in 514 counties. The highest numbers occurred in Muli (54), Kangding (53), Shangri-La (52), and Deqin (37), largely overlapping with richness hotspots and indicating strong spatial concordance between species richness and endemism (Figure 1E). Zayü, Milin, and Motuo recorded 27, 25, and 19 endemic species, with endemism rates of 56.3%, 58.1%, and 46.3%, respectively. In southern Xizang, endemism rates were low in Nyalam (10.0%), Gyirong (13.3%), and Tingri (22.2%), suggesting a transboundary distribution pattern. At the county scale, 266 counties showed 100% endemism, mainly distributed in Sichuan, Shaanxi, Qinghai, Yunnan, Guizhou, Hubei, and Gansu (Figure 1F). High species density was also observed in the Yunnan–Sichuan border region and southeastern Xizang (Figure 2), further supporting the Hengduan Mountains and adjacent regions as key centers of distribution and endemism for *Primula* in China.

### 2.2. Parameter Optimization and Model Validation

**Figure 3:**
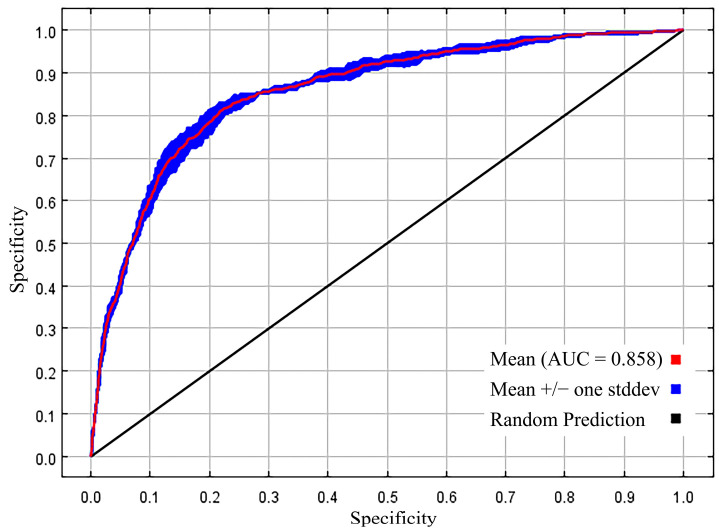
ROC curve of MaxEnt model prediction for *Primula* (mean test AUC over 10 repeated runs: 0.858, standard deviation: 0.003).

Based on 550 filtered occurrence records and 12 environmental variables, model parameter optimization was conducted using the Kuenm package. The optimal parameter combination was identified as a feature combination (FC) of LPT (linear, product, and threshold features) and a regularization multiplier (RM) of 0.9, corresponding to the minimum corrected Akaike Information Criterion (AICc) value. Using these optimized parameters, the MaxEnt model was run with 10 replicated cross-validations. Receiver operating characteristic (ROC) analysis yielded a mean AUC value of 0.858 (Figure 3), indicating good predictive accuracy and model reliability.

### 2.3. Environmental Variables Influencing the Distribution of Primula

**Figure 4:**
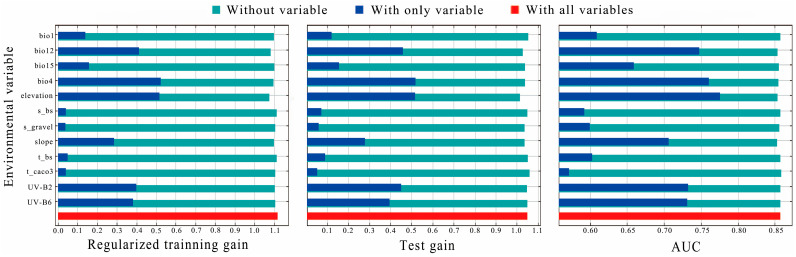
Jackknife test for evaluating major environmental factors.

Environmental variables influencing the distribution of wild *Primula* were evaluated using percentage contribution, permutation importance, and Jackknife tests based on the MaxEnt model. The most influential predictors were bio4 and elevation, with contribution rates of 39.8% and 27.1%, respectively, together accounting for 66.9% of the total contribution. Additional variables included bio1 (11.1%), bio12 (8.7%), slope (3.1%), and bio15 (2.6%), and the combined contribution of these six variables reached 92.4% (Table 1). The Jackknife test showed that bio4, bio12, elevation, slope, UV-B6, and UV-B2 exhibited the highest independent explanatory power, with the greatest gains in training, test, and AUC performance when used individually (Figure 4). Integrating contribution and Jackknife results, seven variables (bio4, bio1, bio12, elevation, slope, UV-B6, and UV-B2) were identified as the dominant environmental factors determining the geographic distribution of *Primula*. Among these, bio4 showed the highest contribution and permutation importance (39.8% and 37.2%, respectively), indicating that temperature seasonality is the primary environmental constraint on its distribution. Elevation showed relatively high contribution (27.1%) but lower permutation importance (16.0%), suggesting a degree of shared information with other environmental variables.

### 2.4. Response Curves of Dominant Environmental Variables

**Figure 5:**
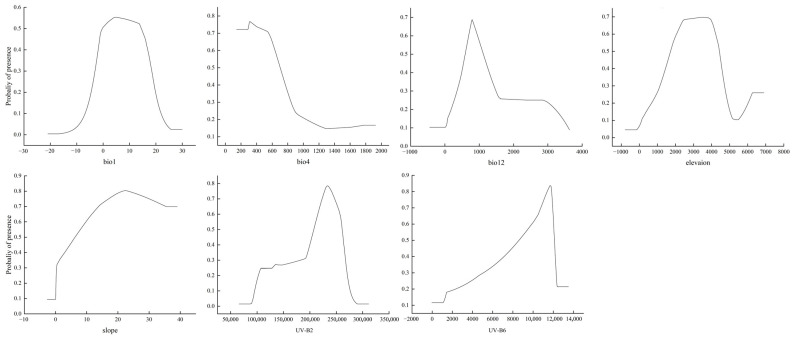
Response curves of dominant environmental factors.

Response curves of the seven dominant environmental variables revealed the suitable environmental thresholds for wild *Primula* (Figure 5), with a habitat suitability probability > 0.5 defined as a suitable habitat. The suitable range of annual mean temperature (bio1) was −0.27–14.44 °C, with maximum suitability at 4.92 °C (probability = 0.55). Temperature seasonality (bio4) ranged from 141.60 to 702.01, with peak suitability at 312.94 (probability = 0.77), whereas annual precipitation (bio12) showed a suitable range of 591.86–1118.67 mm and an optimum at 794.90 mm (probability = 0.69). Suitable elevations ranged from 1733.39 to 4432.44 m, with maximum suitability occurring at 3509.68 m (probability = 0.70). Suitable habitat conditions were reached when slope exceeded 6.24°, with peak suitability at 22.27° (probability = 0.80). Ultraviolet radiation variables also exhibited clear suitability thresholds. The suitable range of UV-B2 was 207,657.02–261,226.47 J m^−2^ d^−1^, with peak suitability at 233,421.37 J m^−2^ d^−1^ (probability = 0.78), whereas UV-B6 ranged from 8606.83 to 12,115.10 J m^−2^ d^−1^, with the highest suitability at 11,708.74 J m^−2^ d^−1^ (probability = 0.84).

### 2.5. Distribution of Suitable Habitats for Primula Under Current and Future Climate Conditions

**Figure 6:**
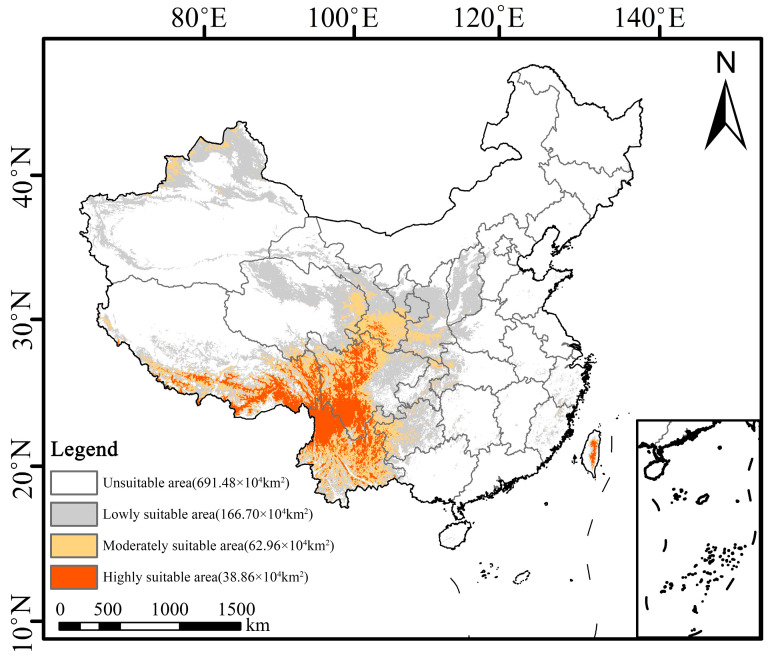
Potential habitat areas of *Primula* in China under current climatic conditions.

**Figure 7:**
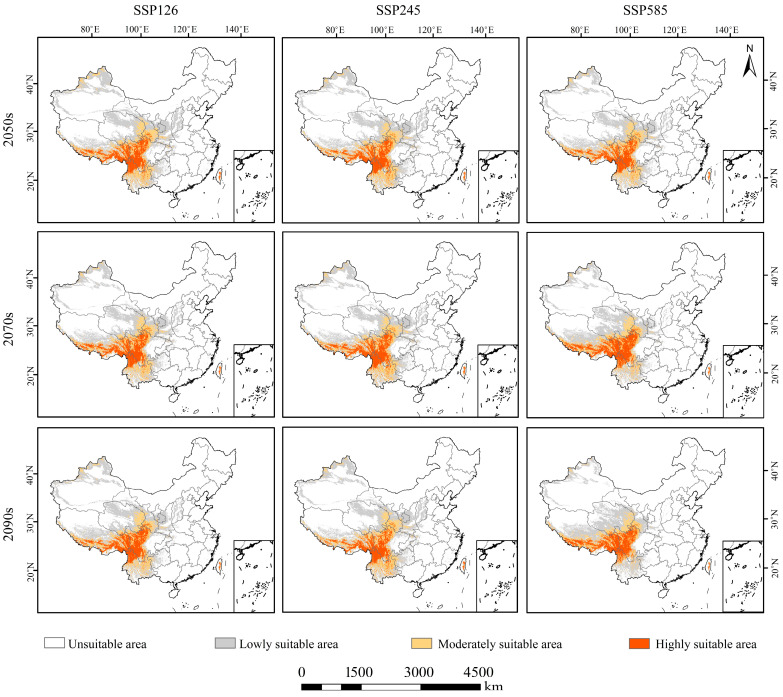
Projected distribution of suitable areas for *Primula* under future climate scenarios.

Under current climatic conditions, suitable habitats for wild *Primula* were mainly distributed in southwestern China, Taiwan, the Loess Plateau, the southeastern Qinghai–Xizang Plateau, and northern Xinjiang (Figure 6). The total suitable area was 268.52 × 10^4^ km^2^, including 38.86 × 10^4^ km^2^ of highly suitable habitat, 62.96 × 10^4^ km^2^ of moderately suitable habitat, and 166.70 × 10^4^ km^2^ of low-suitability habitat. Highly suitable regions were primarily concentrated in the Hengduan Mountains, the southeastern Qinghai–Xizang Plateau, and the Central Mountain Range of Taiwan. Under future climate scenarios, the total suitable area of *Primula* generally declined relative to the current climate (Figure 7). Under the SSP126 scenario, the total suitable area decreased by 9.45%, 10.97%, and 9.01% in the 2050s, 2070s, and 2090s, respectively. Low-suitability habitats showed the greatest reductions, decreasing by 11.47%, 14.95%, and 11.13%, respectively, whereas highly suitable habitats expanded locally in the 2070s, reaching 40.53 × 10^4^ km^2^. Under the SSP245 scenario, the total suitable area decreased by 1.82%, 10.98%, and 7.80% across the three future periods, respectively. The most pronounced contraction of low-suitability habitats occurred in the 2070s (−14.06%), accompanied by an 11.61% reduction in moderately suitable habitats. In contrast, highly suitable habitats remained expanded across all three periods, reaching 41.57 × 10^4^ km^2^, 40.12 × 10^4^ km^2^, and 42.25 × 10^4^ km^2^ in the 2050s, 2070s, and 2090s, respectively. Under the high-emission SSP585 scenario, the total suitable area decreased by 13.13%, 14.34%, and 20.73% across the three future periods, respectively. Low-suitability habitats showed the greatest decline in the 2090s (−25.86%), while moderately suitable habitats decreased by 24.78% during the same period. Highly suitable habitats expanded in the 2070s and 2090s, reaching 46.07 × 10^4^ km^2^ and 41.91 × 10^4^ km^2^, respectively. Overall, low-suitability habitats showed substantial contraction under all future climate scenarios, whereas moderately suitable habitats fluctuated over time. In contrast, highly suitable regions remained comparatively stable and expanded locally during certain periods, particularly in the Hengduan Mountains, the southeastern Qinghai–Xizang Plateau, and the Central Mountain Range of Taiwan. Habitat changes were relatively moderate under SSP126 but became increasingly pronounced under the SSP585 scenario, indicating that *Primula* is more sensitive to high-emission climate conditions. Although highly suitable habitats remained relatively stable, the risk of habitat loss in low-suitability regions increased markedly.

### 2.6. Dynamic Changes in Suitable Habitats of Primula Under Future Climate Scenarios

**Figure 8:**
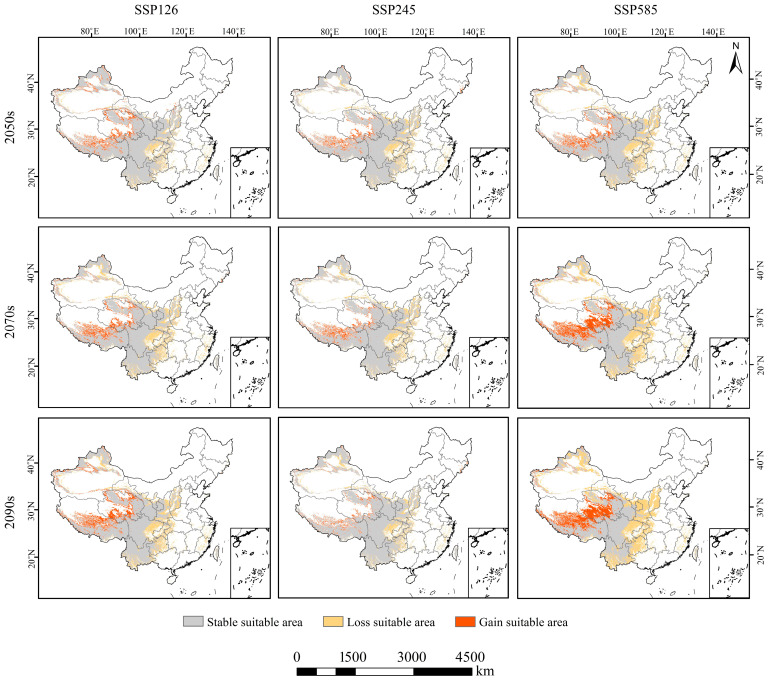
Dynamic changes in suitable habitats for *Primula* under future climate scenarios. The white area represents unsuitable area.

Compared with current suitable habitats of *Primula*, all future climate scenarios exhibited a consistent pattern in which habitat loss exceeded habitat gain, and this imbalance became more pronounced with increasing radiative forcing and over time (Figure 8). Under the SSP126 scenario, habitat loss was 41.40 × 10^4^ km^2^ and 46.19 × 10^4^ km^2^ in the 2050s and 2070s, respectively, whereas habitat gain was 16.04 × 10^4^ km^2^ and 16.73 × 10^4^ km^2^. By the 2090s, habitat loss decreased to 37.78 × 10^4^ km^2^, while habitat gain decreased to 13.59 × 10^4^ km^2^, indicating a relatively moderate net reduction. Under the SSP245 scenario, habitat loss increased from 30.86 × 10^4^ km^2^ in the 2050s to 54.05 × 10^4^ km^2^ in the 2070s and 56.63 × 10^4^ km^2^ in the 2090s. Habitat gain was 25.98 × 10^4^ km^2^, 24.57 × 10^4^ km^2^, and 35.68 × 10^4^ km^2^ in the three corresponding periods. The smallest net habitat loss occurred in the 2050s (4.87 × 10^4^ km^2^), corresponding to a 1.82% reduction in total suitable habitat area, representing the most stable period among all scenarios. Under the SSP585 scenario, habitat loss increased markedly from 57.77 × 10^4^ km^2^ in the 2050s to 83.11 × 10^4^ km^2^ in the 2070s and 107.32 × 10^4^ km^2^ in the 2090s. Although habitat gain increased from 22.51 × 10^4^ km^2^ to 51.66 × 10^4^ km^2^ over the same periods, it remained insufficient to offset habitat loss, indicating a strong net contraction of suitable habitat under high-emission conditions. Spatially, habitat loss exhibited a broadly consistent pattern across all scenarios, primarily concentrated in southern Yunnan, central and eastern Guizhou, northeastern Sichuan, most parts of Shanxi, central and southern Shaanxi, western Hubei, southeastern Chongqing, and central-to-southeastern Xinjiang. Overall, although habitat gain increased under high-emission scenarios, suggesting potential opportunities for upward (altitudinal) or northward range shifts in *Primula* under climate warming, these gains were insufficient to compensate for substantial habitat loss in low-elevation regions, resulting in an overall net reduction in suitable habitat area.

### 2.7. Migration Routes of the Centroid of Suitable Habitats of Primula

**Figure 9:**
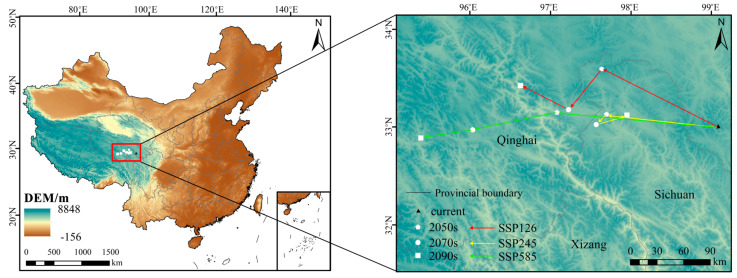
Centroid shifts in suitable habitats for *Primula* under different climate scenarios.

Under all future climate scenarios, the centroid of suitable habitats of *Primula* shows a general westward and northwestward shift, and the migration distance increases with higher-emission scenarios (Figure 9). Under current climatic conditions, the centroid is located in Golog Tibetan Autonomous Prefecture, Qinghai Province (99.19° E, 33.36° N). Under SSP126, the centroid moves 128.12 km northwestward in 2050 to Ganzi Tibetan Autonomous Prefecture, Sichuan Province (97.81° E, 33.37° N). In 2070, it moves 12.82 km southwestward, reaching Yushu Tibetan Autonomous Prefecture, Qinghai Province (97.69° E, 33.25° N), and in 2090 it moves 40.57 km northwestward, still within Yushu Tibetan Autonomous Prefecture (98.06° E, 33.37° N). Under SSP245, the centroid moves 148.41 km northwestward in 2050, reaching Ganzi Tibetan Autonomous Prefecture, Sichuan Province (97.69° E, 33.83° N); in 2070, it moves 40.58 km southwestward but remains within Ganzi Tibetan Autonomous Prefecture (97.34° E, 33.37° N); and in 2090, it moves 68.70 km northwestward but remains within Ganzi Tibetan Autonomous Prefecture (96.72° E, 33.57° N). Under SSP585, the centroid shifts 184.45 km northwestward in 2050 to Yushu (97.20° E, 33.34° N). It then moves 111.95 km southwestward in 2070 (96.20° E, 33.06° N) and further 68.67 km southwestward in 2090 (95.58° E, 32.92° N), still within Yushu. Overall, the centroid consistently shifts westward across scenarios, with a dominant northwestward displacement trend becoming more pronounced under higher-emission pathways. The eastward shift observed in 2090 under SSP126 may be associated with climatic fluctuations or model uncertainty.

### 2.8. Conservation Gap of Primula

**Figure 10:**
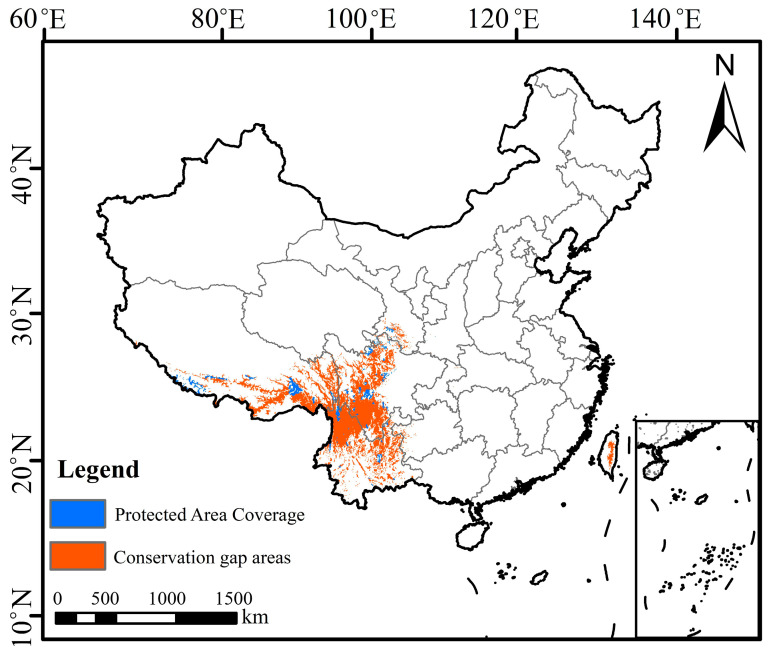
Protection gap of *Primula*.

Within the high-suitability areas of *Primula*, priority conservation areas were identified covering 38.86 × 10^4^ km^2^ (Figure 10), of which only 3.88 × 10^4^ km^2^ (9.99%) fall within existing nature reserves, leaving 90.01% (34.97 × 10^4^ km^2^) unprotected. Spatially, these priority conservation areas are mainly distributed in the Hengduan Mountains, the southeastern Qinghai–Xizang Plateau, and the Central Mountain Range of Taiwan, spanning Yunnan, Sichuan, Xizang, Gansu, Guizhou, and Taiwan. However, only portions of the priority areas in Yunnan, Sichuan, Xizang, and Gansu are currently included within protected areas, while most priority regions remain outside the current conservation network. Overall, these results reveal a substantial conservation gap in the current nature reserve system with respect to highly suitable habitats of *Primula*, highlighting the need for optimization of the existing protected area network.

## 3. Discussion

### 3.1. Dominant Environmental Variables Affecting the Distribution of Primula

The geographic distribution of species is jointly shaped by environmental and biotic factors [53], among which environmental variables are regarded as key determinants constraining the spatiotemporal distribution of endangered species [54,55]. In the present study, the MaxEnt model combined with the Jackknife method was employed to identify the dominant environmental variables affecting the distribution of *Primula*. Following correlation analysis and contribution-based screening of 60 environmental variables, 12 variables representing climatic, soil, and topographic conditions were retained for model construction. The Jackknife test revealed that bio4, elevation, bio1, bio12, slope, UV-B6, and UV-B2 were the principal limiting factors affecting the distribution of *Primula*. Among these variables, temperature-related factors (bio4 and bio1) jointly accounted for 50.9% of the total contribution, indicating that temperature plays a dominant role in shaping the distribution pattern of *Primula*. In particular, bio4 was identified as the most influential variable (39.8%), followed by elevation (27.1%) and bio12 (8.7%). Previous studies have shown that the growth and reproduction of alpine plants are strongly constrained by temperature and resource availability, including soil nutrients, precipitation, and solar radiation [56,57]. The distribution of *Primula* is also highly sensitive to temperature and moisture conditions [58]. For example, Jiang et al. reported that bio6 and bio19 were the dominant factors affecting the distribution of *P. filchnerae* in Shaanxi Province, with a combined contribution exceeding 65% [35]. Li et al. identified annual precipitation as the most important determinant limiting the distribution of *P. crystallophlomis* (46.2%), followed by temperature seasonality (29.2%) [49]. T. Yang further demonstrated that temperature and precipitation-related variables strongly influenced the geographic distribution of *Lysimachia* within Primulaceae, with bio14, bio12, bio4, and bio6 identified as important climatic factors [59]. Temperature seasonality (bio4) reflects the magnitude of annual temperature fluctuations, with higher values indicating greater seasonal variation and lower climatic stability. The persistence of *Primula* in regions characterized by relatively high bio4 values suggests strong tolerance to seasonal temperature variability, likely reflecting long-term ecological adaptation to alpine environments. Similar patterns have also been reported in other plant species. Tang et al. found that bio4 contributed 25.2% to the distribution of *Epimedium koreanum*, second only to bio18 [60]. Mi et al. identified bio4 as the primary limiting factor affecting the distribution of *Thymus asiaticus* (23.9%) [61]. Although the contribution of bio4 in these studies was lower than that observed in the present study, it remained the dominant limiting factor. Previous studies have further demonstrated that alternating temperature regimes (e.g., 15/5 °C) significantly regulate seed germination in certain *Primula* species [62], while the combination of dry storage and cold stratification can enhance germination rates [63]. These findings suggest that moderate seasonal temperature fluctuations may facilitate the release of physiological seed dormancy through the combined effects of cold stratification and alternating warm-cold cycles. Such temperature-dependent germination mechanisms further reflect the ecological adaptation of *Primula* to seasonal climatic fluctuations in alpine environments.

In addition to climatic variables, elevation (27.1%) and slope (3.1%) were identified as important topographic factors influencing the distribution of *Primula*. Species richness is mainly concentrated at elevations between 3000 and 4000 m, corresponding to transitional zones between coniferous forests and alpine shrub–meadow ecosystems that provide diverse humid microhabitats [64]. Chen et al. similarly identified elevation (16.6%) as a key topographic factor affecting *P.* Sect. *Proliferae* [48], although its contribution was lower than that observed in the present study, possibly because *Primula* occupies a broader elevational range. Notably, UV-B6 (1.80%) and UV-B2 (1.40%) were also identified as important predictors of *Primula* distribution. UV-B radiation plays an important role in the morphological evolution of alpine plants [65]. Moderate UV-B exposure can promote plant growth and physiological performance by enhancing secondary metabolite accumulation, improving resistance to biotic and abiotic stresses, enhancing photosynthetic capacity, and promoting flowering, whereas excessive exposure may exert adverse effects [66,67,68,69,70,71]. Consequently, UV-B radiation has also been recognized as an important predictor for certain herbaceous species. For example, Xie et al. found that UV-B2 was the primary factor influencing the distribution of *Chrysanthemum indicum*, with a contribution rate of 44.5%, and further suggested that UV-B radiation substantially affects the geographic distribution of plants in middle and high-latitude regions [72].

### 3.2. Spatial Dynamics and Centroid Shifts in Potential Suitable Habitats of Primula Under Different Climate Scenarios

Our projections reveal a consistent net contraction of suitable habitats for *Primula* under future climate scenarios, characterized by a distinctive asymmetric pattern of core stability at high elevations and peripheral erosion at lower elevations. This suggests that marginal populations, particularly those at the warmer edges of the current distribution, are disproportionately vulnerable to climate warming. The observed upslope shift—driven by warming enhancing the climatic suitability of high-elevation zones—aligns with the recognized buffering effect of complex topography in the Hengduan Mountains, which facilitates altitudinal range adjustments toward the Qinghai–Xizang Plateau and mitigates extensive habitat loss [73]. This mechanism corroborates the identification of northwestern Yunnan, western Sichuan, and southeastern Tibet as relatively stable refugia for alpine herbs under climate change [74]. Spatially, the contraction is primarily driven by substantial losses at the current distributional margins, including southern Yunnan, central-eastern Guizhou, the Sichuan Basin periphery, and central Xinjiang. Although novel suitable patches emerge in high-elevation areas, their limited extent and high fragmentation render them insufficient to counterbalance the losses in low-elevation margins, resulting in a net habitat reduction. This pattern of net contraction with upslope retreat mirrors findings for *Lancea tibetica*, which is projected to lose 40–60% of its suitable habitat under similar climatic forcing [75]. The consistent westward and northwestward displacement of the centroid across all scenarios—with increasing migration distances under higher-emission pathways—further reinforces this trend. Such directional shifts are congruent with the general biogeographic expectation of poleward and upward range migrations, as documented in Himalayan *Meconopsis* species [76] and *Valeriana wallichii* [77], both exhibiting analogous north–westward shifts. The Hengduan Mountains and southeastern Tibetan Plateau are likely to serve as climatic refugia for *Primula*-defined as areas where species persist during unfavorable climatic periods, either as continuous distributions or as isolated populations [78]. This inference is strongly supported by phylogeographic evidence confirming that multiple *Primula* species, including *P. ovalifolia* [79] and *P. secundiflora* [80], persisted in glacial refugia shaped by the region’s complex topography. However, the effective realization of these refugial potentials is critically challenged by the inherent dispersal limitations of the genus. *Primula* species lack specialized long-distance dispersal structures, relying primarily on short-range seed dispersal [81], with occasional transport via floods, small mammals, and ants permitting only negligible range extension [82,83]. Moreover, the parallel mountain ranges and deep valley systems act as physical barriers that severely restrict gene flow and population connectivity [84]. Consequently, despite the existence of climatically suitable northwestern habitats, the actual migration of *Primula* toward these areas is likely to be constrained by fragmented connectivity along the migration pathways. From a conservation perspective, these findings underscore the urgent need for targeted interventions. Priority should be accorded to in situ protection of the core refugial habitats in the Hengduan Mountains and southeastern Tibetan Plateau, where climatic stability is projected to persist. In parallel, establishing ecological corridors along predicted migration routes is essential to maintain landscape connectivity, mitigate habitat fragmentation, and ultimately support the long-term persistence of *Primula* populations under ongoing climate change.

### 3.3. Distribution Pattern and Conservation Gaps of Primula

Geographic distribution reflects the long-term interplay between species and their environment [85]. This distributional pattern reveals that *Primula* is widely but unevenly distributed across China, with a pronounced concentration in the southwestern Hengduan Mountains and the eastern Himalayas. Sichuan, Yunnan, and the Xizang Autonomous Region harbor the highest species richness nationwide; at the county level, Shangri-La City, Muli Tibetan Autonomous County, and Kangding City represent the peak centers of species richness. This spatial configuration corroborates Richards’ view that the mountains of southwestern China and the Himalayas (shared with Nepal, Bhutan, and other countries) collectively constitute the modern distribution center of *Primula* [86]. The spatial pattern of endemism, however, diverges notably from that of species richness. Hotspots of species differentiation extend beyond the species-rich southwestern core region; Hubei, Chongqing, Guizhou, and Hunan in central China, along with portions of the southwest, also exhibit relatively high endemism rates. Remarkably, 266 counties exhibit an endemism rate of 100%, a striking figure that underscores the role of central China and parts of the southwest as important centers of endemism differentiation. Such extremely high rates may reflect the combined effects of localized microhabitat specialization, historical isolation, and potentially uneven sampling intensity across regions. In contrast, southern border counties of Xizang (e.g., Nyalam, with an endemism rate of only 10.0%; Gyirong, 13.3%; and Tingri, 22.2%) and eastern Himalayan counties such as Zayü, Miling, and Motuo show moderate endemism rates, likely attributable to extensive floristic exchanges with neighboring countries. Furthermore, the eastern margin of the Qinghai–Xizang Plateau—stretching from northern Myanmar along the Himalayas to northern India—is also recognized as a global hotspot for *Primula* distribution [81], resulting in a pronounced transboundary distribution pattern of the genus in this region. The conservation significance of this distributional pattern is further underscored by a substantial protection gap: 90.01% of the 38.86 × 10^4^ km^2^ of highly suitable habitat lies outside the existing nature reserve system. These gaps are concentrated in regions of high species richness and endemism, including Yunnan, Sichuan, Xizang, Gansu, and Guizhou, highlighting a stark mismatch between conservation priority and actual protection coverage. This gap is shaped by a combination of factors, including the spatially clustered distribution of *Primula*, marked provincial disparities in reserve allocation, and the broader “west-poor-east-rich, west-clustered-east-scattered” characteristics of China’s protected area network [87]. Additionally, the core habitats of *Primula* are predominantly located in mid- to high-elevation vegetation transition zones or alpine shrub meadows; given its preference for cool, humid conditions [88], the genus is largely concentrated within the aforementioned conservation gap areas. The specialized adaptation of *Primula* to alpine environments, coupled with its limited dispersal capacity [81], renders the genus particularly vulnerable to future climate warming, which is projected to drive its suitable habitats to contract northwestward or toward higher altitudes. With the vast majority of its critical habitats currently unprotected, *Primula* is exposed to the dual, compounding threats of habitat degradation and climate-induced range contraction. In the absence of timely intervention, the synergistic effects of these pressures may accelerate population declines and elevate extinction risks for many narrow-ranged species. To mitigate these compounding pressures, targeted conservation actions are urgently required. Priority should be accorded to establishing new protected areas or conservation micro-reserves in hotspot gap regions, including southwestern Sichuan, northwestern Yunnan, southeastern Xizang, and southern Gansu. This approach has proven effective in alleviating habitat fragmentation of wild plants [89] and has yielded successful outcomes in conservation micro-reserve construction in Yunnan, China [90]. Concurrently, the planning of the reserve network must fully integrate the predicted climate-driven migration corridors of *Primula* to enhance the coverage and connectivity of critical habitats, thereby ensuring the long-term persistence of this important alpine plant genus under ongoing climate change.

## 4. Materials and Methods

### 4.1. Data and Materials

#### 4.1.1. Geographic Distribution Data of *Primula*

**Figure 11:**
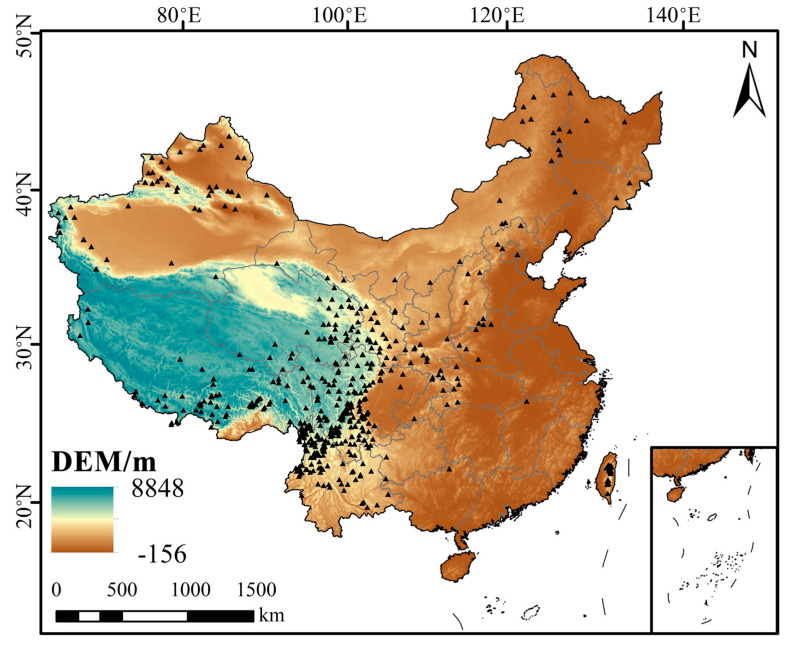
Distribution points of *Primula*.

Occurrence data for *Primula* in China were compiled from four complementary sources: (1) the Flora of China (Volume 15), regional floras, and related plant checklists; (2) 44 monographs and books, including Diversity and Geographical Distribution of *Primula* in China and Vascular Plants of the Qinghai–Xizang Plateau and Their Eco-geographical Distribution; (3) 118 academic papers (including master’s and doctoral theses) reporting new taxa or new distribution records of *Primula*; and (4) specimen records from the National Specimen Information Infrastructure (NSII, http://www.nsii.org.cn/, accessed on 5 March 2026) and the Chinese Virtual Herbarium (CVH, https://www.cvh.ac.cn/, accessed on 5 March 2026), totaling 3256 records. Geographic coordinates for all occurrences were obtained from three sources: the Global Biodiversity Information Facility (GBIF, http://www.gbif.org, accessed on 5 March 2026), the Chinese Virtual Herbarium (CVH, http://www.cvh.ac.cn, accessed on 5 March 2026), and published literature. For records lacking explicit coordinate information in the literature, coordinates were extracted based on detailed locality descriptions using the georeferencing function of the R package ggmap (version 3.0.0). A total of 7647 coordinate records were initially collected. To reduce spatial autocorrelation and mitigate the risk of model overfitting and inflated performance estimates, a common preprocessing step [91] was applied using ENMtool (version 1.4.0): when multiple occurrence points fell within the same grid cell (spatial resolution: 2.5 arc-minutes), only one point was retained. After this filtering procedure, a final set of 550 spatially independent occurrence points (Figure 11) was obtained for subsequent species distribution modeling.

#### 4.1.2. Sources and Selection of Environmental Data

Four groups of environmental variables were compiled to predict the suitable habitats of *Primula*: (1) 19 bioclimatic variables (bio1–bio19) from WorldClim version 2.1 (https://www.worldclim.org/, accessed on 1 April 2026), with a spatial resolution of 2.5 arc-minutes. The temporal range covered the current period (1970–2000, representing the 1980s) and three future periods: 2041–2060 (2050s), 2061–2080 (2070s), and 2081–2100 (2090s). Future climate projections were obtained under three Shared Socioeconomic Pathways (SSP126, SSP245, and SSP585) using the BCC-CSM2-MR climate model, which was selected for its demonstrated robust performance in simulating regional climate dynamics over China [92,93,94]; (2) three topographic variables (slope, aspect, and elevation) extracted from the Digital Elevation Model (DEM, http://www.gscloud.cn/, accessed on 1 April 2026) and processed using ArcGIS spatial analysis tools; (3) 32 soil variables from the Harmonized World Soil Database (HWSD version 2.0, https://www.fao.org/soils-portal/, accessed on 1 April 2026); and (4) six ultraviolet (UV) radiation variables from the Global UV Database (https://www.ufz.de/gluv/, accessed on 1 April 2026). In total, 60 environmental predictor variables were used for initial modeling. Before modeling, all variables were uniformly preprocessed: raster layers were converted to ASCII format in ArcGIS 10.8 and standardized to the same geographic coordinate system (GCS_WGS_1984), spatial resolution (2.5 arc-minutes), and study area boundary. To reduce the effects of multicollinearity on the MaxEnt model, a two-step variable screening procedure was applied. First, an initial MaxEnt model was run to calculate the percent contribution of each variable. Second, Pearson correlation coefficients were calculated based on the values extracted from all occurrence points at their corresponding grid cells; for any variable pair with an absolute correlation coefficient > 0.8, the variable with the lower contribution was excluded. Through this screening process, 12 variables with low multicollinearity and high contribution were selected from the original 60 for the final modeling.

### 4.2. Species Geographic Distribution and Density Analysis

Species data were standardized and carefully validated using the Flora of China (Volume 15) and the Catalogue of Life China (http://www.sp2000.org.cn/CoLChina, accessed on 1 April 2026). The conservation status of each species was checked against the China Biodiversity Red List [32]. Occurrence locality names were verified one by one using the query tools and toponym conversion function of the National Specimen Information Infrastructure (NSII, http://www.nsii.org.cn/, accessed on 5 March 2026) and the Chinese Virtual Herbarium, together with the standard map of China published by the Ministry of Natural Resources (http://bzdt.ch.mnr.gov.cn/, accessed on 1 April 2026). Distribution data of wild *Primula* in China were then spatially joined with administrative boundaries using ArcGIS 10.2, and the distribution pattern was classified into five classes using the natural breaks (Jenks) method [95]. Using county-level administrative units as evaluation units, the number of wild *Primula* species within each unit was counted. To eliminate the effect of area differences among units on species density analysis, the species–area relationship was applied using the following formula:

ρs = log(Ns)/log(A)

where Ns is the number of wild *Primula* species in a given county, A is the area of that county, and ρs is the species distribution density.

### 4.3. Establishment and Evaluation of Species Distribution Models

To improve the accuracy and stability of habitat suitability predictions, the Kuenm package in R was used to optimize the feature classes (FC) and regularization multiplier (RM) parameters in the MaxEnt model [96]. The performance of the MaxEnt model is primarily influenced by these two parameter types. FC determines the functional form of the species–environment response relationship, including five basic types: linear (L), quadratic (Q), hinge (H), product (P), and threshold (T). All non-empty combinations of these features (31 in total) were considered. RM controls model complexity to prevent overfitting and was set to range from 0.1 to 4.0 at 0.1 intervals (40 values in total) [97]. Using the optimized parameter combination, species occurrence data and environmental variables were fed into the MaxEnt model for habitat suitability modeling and prediction. For model validation, 25% of the occurrence records were randomly selected as the test dataset for each species, with the remaining 75% used as the training dataset [98]. To reduce uncertainty from random partitioning, the model was run 10 times under each parameter combination with the “create response curves” option enabled. The final output was the mean of the 10 replicate runs, and the standard deviation was calculated simultaneously to assess model stability. The continuous probability distribution maps were then converted into binary presence/absence maps using the threshold that maximizes the sum of training sensitivity and specificity, which robustly balances omission rate and predicted area. The jackknife test was used to evaluate the importance of each environmental variable and its independent contribution to model gain [99]. This method identifies the key variables that contribute most to predictions by sequentially omitting each variable, rebuilding the model, and comparing the resulting changes in model performance. Finally, model performance was evaluated using the area under the receiver operating characteristic curve (AUC), which ranges from 0.5 to 1.0, where 0.5 indicates performance equivalent to random guessing and 1.0 indicates perfect prediction. According to conventional interpretation criteria: AUC < 0.6 indicates an invalid model; 0.6–0.7 indicates a relatively poor model; 0.7–0.8 is acceptable; 0.8–0.9 is good; and 0.9–1.0 is excellent [100].

### 4.4. Classification of Suitable Habitats and Dynamic Changes in Spatial Distribution Patterns

Based on the predicted occurrence probabilities (range 0–1) from the MaxEnt model, the study area was classified into four habitat suitability classes using the natural breaks (Jenks) method in ArcGIS: unsuitable (<0.20), low suitability (0.20–0.40), medium suitability (0.40–0.60), and high suitability (>0.60). Corresponding suitability distribution maps were generated [101]. The number of grid cells per class was calculated using the Raster Calculator and Extract by Attributes tools. The environmental raster data had a spatial resolution of 2.5 arc-minutes (approx. 4.5 km × 4.5 km), with each cell covering about 20.25 km^2^. Thus, the total area (km^2^) for each class was calculated as the product of its cell count and the unit cell area. To assess the response of *Primula* geographic suitability to future climate scenarios, overlay analysis was performed on binary suitability maps (suitable = 1, unsuitable = 0) under current and future climate scenarios, and a habitat change matrix was derived. This matrix classified habitat dynamics into four types: 0–1 = expansion (newly suitable areas); 1–0 = contraction (lost suitable areas); 1–1 = persistent suitability (stable areas); and 0–0 = persistent unsuitability [102]. The distribution centroid was defined as the geometric center of a species’ distribution within a given spatial extent, reflecting its spatial aggregation or migration trend [103]. Because the boundaries of suitable distribution areas of *Primula* are often irregular, centroid analysis provides an effective means of quantifying spatial displacement. Accordingly, the SDMtoolbox for ArcGIS (version 2.4; Brown, 2014) was used to calculate centroid positions of suitable areas under current and future climate scenarios. The displacement distance (km) between centroids was then calculated using Euclidean distance, and the displacement direction angle was recorded to evaluate spatial migration patterns of *Primula* under climate change.

### 4.5. Conservation Gap Analysis of Primula

To identify priority conservation areas for *Primula* under climate change and evaluate the effectiveness of existing nature reserves, high-suitability areas (occurrence probability > 0.60) from the MaxEnt model were used to define priority conservation habitats. Using the boundaries of existing national and provincial nature reserves in China as a spatial constraint layer, the Extract by Mask tool was used to identify areas of overlap between high-suitability habitats of *Primula* and nature reserves for the current period and three future periods (2050s, 2070s, 2090s). These overlapping areas were defined as the priority conservation areas for each period. Consequently, the total area (km^2^) and its percentage change across climate periods were quantified to assess potential climate change impacts on protected areas. Furthermore, spatial overlay analysis was used to compare the spatial dynamics of priority conservation areas across different periods (expansion, contraction, and stability), thus allowing for assessment of the potential impacts of climate change on their spatial pattern.

## 5. Conclusions

This study assesses the distribution patterns, climate-change vulnerability, and conservation priorities of *Primula* in China using MaxEnt modeling integrated with topographic, climatic, and soil variables. The genus exhibits a distribution centered in the Hengduan Mountains and eastern Himalayas, with central China and parts of the southwest as secondary endemism hotspots—a macro-geographic pattern of “core aggregation with peripheral independent differentiation.” The optimized MaxEnt model (AUC = 0.858) identified temperature seasonality (bio4, 39.8% contribution) and elevation (27.1%) as the dominant distributional constraints. Future climate projections indicate continuous contraction of total suitable habitat, with marginal low-suitability areas facing disproportionate loss and the distribution centroid shifting westward and northwestward—trends that intensify under higher-emission scenarios. In contrast, high-suitability habitats in the Hengduan Mountains and southeastern Qinghai–Xizang Plateau are expected to remain relatively stable, serving as potential climatic refugia. However, >90% of priority conservation areas currently lack protection. Targeted actions are urgently needed: new protected areas or micro-reserves should be established in hotspot gaps (southwestern Sichuan, northwestern Yunnan, southeastern Xizang, and southern Gansu), and predicted migration corridors must be integrated into reserve network planning to enhance connectivity and mitigate fragmentation threats to the genus. Several limitations warrant acknowledgment. Projections rely on a single climate model (BCC-CSM2-MR); multi-GCM ensembles would improve robustness. Species occurrence data may suffer from collection biases and spatial autocorrelation. MaxEnt does not account for interspecific competition or dispersal limitations, and our genus-level approach may mask niche differences among species. Future studies should integrate ensemble climate projections and refined species-level occurrence data. Despite these caveats, our findings inform conservation planning by identifying core refugia, protection gaps, and migration corridors. We advocate urgent implementation of the proposed strategies to safeguard this alpine genus against the compounding threats of climate change and habitat degradation, ensuring its long-term viability in the wild.

## Figures and Tables

**Table 1:** Parameters of environmental variables.

Code	Environmental Variable	Contribution Rate/%	Permutation Importance/%
bio4	temperature seasonality	39.80	37.20
elevation	elevation	27.10	16.00
bio1	annual mean temperature	11.10	11.70
bio12	annual precipitation	8.70	10.50
slope	slope	3.10	6.30
bio15	precipitation seasonality	2.60	3.80
s_gravel	subsoil stone volume percentage	1.80	2.40
UV-B6	sum of monthly mean UV-B during lowest quarte	1.80	4.60
UV-B2	UV-B seasonality	1.40	3.40
t_caco3	top soil layer carbonate	1.30	3.00
t_bs	top soil base saturation	1.10	0.80
s_bs	subsoil soil base saturation	0.20	0.30

## Data Availability

The original contributions presented in this study are included in the article. Further inquiries can be directed to the corresponding author.

## References

[1] Pecl G.T., Araújo M.B., Bell J.D., Blanchard J., Bonebrake T.C., Chen I.-C., Clark T.D., Colwell R.K., Danielsen F., Evengård B. (2017). Biodiversity redistribution under climate change: Impacts on ecosystems and human well-being. Science.

[2] Franks S.J., Weber J.J., Aitken S.N. (2014). Evolutionary and plastic responses to climate change in terrestrial plant populations. Evol. Appl..

[3] Bellard C., Bertelsmeier C., Leadley P., Thuiller W., Courchamp F. (2012). Impacts of climate change on the future of biodiversity. Ecol. Lett..

[4] Lenoir J., Svenning J.-C. (2015). Climate-related range shifts—A global multidimensional synthesis and new research directions. Ecography.

[5] Masson-Delmotte V., Zhai P., Pörtner H.-O., Roberts D., Skea J., Shukla P.R., Pirani A., Moufouma-Okia W., Péan C., Pidcock R. (2022). Global Warming of 1.5 °C: IPCC Special Report on Impacts of Global Warming of 1.5 °C above Pre-Industrial Levels in Context of Strengthening Response to Climate Change, Sustainable Development, and Efforts to Eradicate Poverty.

[6] Overland J., Dunlea E., Box J.E., Corell R., Forsius M., Kattsov V., Olsen M.S., Pawlak J., Reiersen L.O., Wang M. (2019). The urgency of Arctic change. Polar Sci..

[7] Akyol A., Örücü Ö.K., Arslan E.S. (2021). Climate change effect on the potential distribution of the Anatolian chestnut (*Castanea sativa* Mill.) in the coming century in Turkey. Theor. Appl. Climatol..

[8] Editorial Committee of the Fourth National Assessment Report on Climate Change (2022). The Fourth National Assessment Report on Climate Change: Scientific Basis.

[9] Li D., Wei J., Xu Z., He L., Xu D., Shen D. (2024). Prediction and Analysis of Potential Suitable Habitats for *Rhododendron huadingense* Based on MaxEnt Model. J. Zhejiang For. Sci. Technol..

[10] Zhang H., Zhu Y., Ma Z., He J., Guo C., Zhou Q., Song L. (2025). Simulating the impact of climate change on the suitable area for cotton in Xinjiang based on SDMs model. Ind. Crops Prod..

[11] Zhao Z., Xiao N., Shen M., Li J. (2022). Comparison between optimized MaxEnt and random forest modeling in predicting potential distribution: A case study with *Quasipaa boulengeri* in China. Sci. Total Environ..

[12] Yang Z., Zhang D., Wei Y., Guo L., Cheng L. (2026). Invasion risk of *Galinsoga quadriradiata* on the Qinghai-Tibet Plateau under climate change. Arid Zone Res..

[13] Cai R.Z.X., Liu Y.P., Su X., Qu R.J., Wei K.Y., Feng X., Sun C.L., Zhang K.Q. (2026). Prediction of potential suitable areas for the endemic genus *Orinus* on the Qinghai-Tibet Plateau under climate change. Acta Agrest. Sin..

[14] Tang J., Xuan Z., Yin X., Yu Y., Yan S., Li H., Huang X. (2026). Geographical distribution simulation and priority conservation area identification for the extremely small population plant Magnolia sinensis. J. Southwest For. Univ..

[15] Yang J., Tian Y., Zhou H., Guo T., Yi X., Chen J. (2026). Potential suitable areas of the invasive alien plant Leucaena leucocephala in China under climate change scenarios. J. China West Norm. Univ..

[16] Wang Y., Xie L., Zhou X., Chen R., Zhao G., Zhang F. (2023). Prediction of the potentially suitable areas of *Leonurus japonicus* in China based on future climate change using the optimized MaxEnt model. Ecol. Evol..

[17] Aidoo O.F., Souza P.G.C., Silva R.S., Júnior P.A.S., Picanço M.C., Osei-Owusu J., Sétamou M., Ekesi S., Borgemeister C. (2022). A machine learning algorithm-based approach (MaxEnt) for predicting invasive potential of *Trioza erytreae* on a global scale. Ecol. Inform..

[18] Lin W., Ren Y., Fan G., Deng M., Liu Y., Zhang Q., Xu X., Huang S., Zhang H., Qi J. (2025). Projecting suitable habitats and prioritizing conservation areas for *Dendrobium shixingense* under climate change. Front. Plant Sci..

[19] Huang L., Yao W., Xiao X., Zhang Y., Chen R., Yang Y., Li Z. (2025). Predicting potentially suitable habitats and analyzing the distribution patterns of the rare and endangered genus *Syndiclis* Hook. f. (*Lauraceae*) in China. Plants.

[20] Wu Y., Yan L., Shen H., Guan R., Ge Q., Huang L., Rohani E.R., Ou J., Han R., Tong X. (2025). Potentially suitable geographical area for *Pulsatilla chinensis* Regel under current and future climatic scenarios based on the MaxEnt model. Front. Plant Sci..

[21] Hu C.M., Chen F.H., Hu C.M. (1990). *Primula*. Flora Reipublicae Popularis Sinicae.

[22] Hu C.M., Kelso S., Wu Z.Y., Raven P.H. (1996). Primulaceae. Flora of China.

[23] POWO. Plants of the World Online. http://powo.science.kew.org/.

[24] Hao G., Hu C.M., Lee N.S. (2002). Circumscriptions and phylogenetic relationships of *Primula* sects. *Auganthus* and *Ranunculoides*: Evidence from nrDNA ITS sequences. J. Integr. Plant Biol..

[25] Lin C., Liu B., Zhao M., Ma K., Ji L. (2025). China Checklist of Higher Plants. Catalogue of Life China: 2025 Annual Checklist.

[26] Gu J., Wang H., Lin H., Jiang X., Shuai T., Wu Z. (2025). *Primula jiangyouensis* (*Primulaceae*), a new species of *Primula* sect. *Auganthus* from Sichuan, China. PhytoKeys.

[27] Shuai T., Lin H., Cai L., Chen Y., Wu Z. (2025). *Primula yanbianensis* (*Primulaceae*), a new species in *Primula* sect. *Cortusoides* from Sichuan, China. PhytoKeys.

[28] Zhang S., Hu Y., Gu J., Hu Y., Si X., Zhang W., Shao J. (2025). Two new species of *Primula* sect. *Auganthus* from Sichuan, China. Ecol. Evol..

[29] Tang S.H., Long Z.X., Li F.W. (2025). *Primula xinjingensis* (*Primulaceae*), a new species from Guizhou, China. PhytoKeys.

[30] Hu Q.M. (1994). On the geographical distribution of Primulaceae. J. Trop. Subtrop. Bot..

[31] Yang G., Zhang J., Mei L., Li X., Long S., Bai X. (2023). A dataset of the diversity and geographical distributions of wild *Primula* L. in China. China Sci. Data.

[32] Ministry of Ecology and Environment of China, Chinese Academy of Sciences China Biodiversity Red List Higher Plants Volume. https://www.mee.gov.cn/xxgk2018/xxgk/xxgk01/202305/W020230522536560832337.pdf.

[33] Colombo P.S., Flamini G., Rodondi G., Giuliani C., Santagostini L., Fico G. (2017). Phytochemistry of European *Primula* species. Phytochemistry.

[34] Schmidt-Lebuhn A.N., de Vos J.M., Keller B., Conti E. (2012). Phylogenetic analysis of *Primula* section *Primula* reveals rampant non-monophyly among morphologically distinct species. Mol. Phylogenet. Evol..

[35] Jiang X., Liu W.-J., Zhu Y.-Z., Cao Y.-T., Yang X.-M., Geng Y., Zhang F.-J., Sun R.-Q., Jia R.-W., Yan C.-L. (2023). Impacts of climate changes on geographic distribution of *Primula filchnerae*, an endangered herb in China. Plants.

[36] Yamamizo C., Hirashima M., Kishimoto S., Ohmiya A. (2011). Carotenoid composition in the yellow and pale green petals of *Primula* species. Bull. Natl. Inst. Flor. Sci..

[37] Schott H.W. (1851). Die Sippen der Österreichischen Primeln.

[38] Pax F. (1889). Monographische Übersicht über die Arten der Gattung *Primula*. Bot. Jahrb. Syst..

[39] Smith W.W., Fletcher H.R. (1949). The genus *Primula*: Sections *Cuneifolia*, *Floribunda*, *Parryi*, and *Auricula*. Trans. R. Soc. Edinb. Earth Sci..

[40] Wendelbo P. (1961). Studies in Primulaceae, a Monograph of the Genus Dionysia.

[41] Halda J.J. (1992). The Genus Primula in Cultivation and the Wild.

[42] Yan H., He C., Peng C., Hu C., Hao G. (2010). Circumscription of *Primula* subgenus *Auganthus* (*Primulaceae*) based on chloroplast DNA sequences. J. Syst. Evol..

[43] Ren G., Conti E., Salamin N. (2015). Phylogeny and biogeography of *Primula* sect. *Armerina*: Implications for plant evolution under climate change and the uplift of the Qinghai-Tibet Plateau. BMC Evol. Biol..

[44] Yan H., Liu Y., Xie X., Zhang C., Hu C., Hao G., Ge X. (2015). DNA barcoding evaluation and its taxonomic implications in the species-rich genus *Primula* L. in China. PLoS ONE.

[45] Stefanis I., Chatzopoulou P., Krigas N., Karioti A. (2023). Exploring the chemical content of *Primula veris* L. subsp. *veris* wild-growing populations along a climate gradient: An HPLC-PDA-MS quality assessment of flowers, leaves and roots for sustainable exploitation. Horticulturae.

[46] Alam F., Din K.M., Sarfraz M., Qudoos A., Malik S. (2024). Genus *Primula* and its role in phytomedicine; a systematic review. Phytomed. Plus.

[47] Wang X., Sun H., Xu Y., Cao F., Wang Y., Ma J., Li J., Liu L., Li P., Zhang X. (2025). Morphological characteristics and identification of key genes regulating distyly morph in *Primula vulgaris*. Agronomy.

[48] Chen Y., Li W., Zhao F., Wu Z., Huang Y. (2024). Simulation of the potential range of sect. *Proliferae* of the genus *Primula* in Primulaceae. J. Yunnan Norm. Univ..

[49] Li A., Zhou H., Luo X., Wang J., Tian J., Fu Z., Xie G., Li L., Hua D. (2025). The influence of climate change on *Primula* sect. *Crystallophlomis* in southwest China. BMC Plant Biol..

[50] Shi T., Mo Z., Wu M., Zhao C. (2022). Phylogeography of medicinal and edible homologous plant *Allium macrostemon*. Bull. Bot. Res..

[51] Wehn S., Johansen L. (2015). The distribution of the endemic plant *Primula scandinavica*, at local and national scales, in changing mountainous environments. Biodiversity.

[52] Li L., Qin H., Lughadha E.N., Zheng Y., Wan H., Plummer J., Howes M.-J.R., Liu H., Jiang Y., Wang T. (2023). Red list assessments of Chinese higher plants. Int. J. Digit. Earth.

[53] Elith J., Leathwick J.R. (2009). Species distribution models: Ecological explanation and prediction across space and time. Annu. Rev. Ecol. Evol. Syst..

[54] Kaye L.A., Walter G.H., Raghu S. (2016). Patchy distribution and varied habitats of *Macrozamia lucida* cycads explained by constancy in a key environmental variable. Aust. J. Bot..

[55] Lü Z., Zhu X., Ye X., Wen G., Jiang T., Lai W., Shi C., Huang Q., Zhang G. (2024). Impacts of climate change on the suitable habitats and spatial migration of *Tetraena mongolica*. Acta Ecol. Sin..

[56] Bloom A.J., Chapin F.S., Mooney H.A. (1985). Resource limitation in plants—An economic analogy. Annu. Rev. Ecol. Syst..

[57] Zhang L., Zhang Y., Zhao X., Huang S., Zhao J., Yang Y. (2014). Effects of different nutrient sources on plasticity of reproductive strategies in a monoecious species, *Sagittaria graminea* (*Alismataceae*). J. Syst. Evol..

[58] Zhou T. (2003). Studies on Introduction and Cultivation of *Primula pulverulenta*. Master’s Thesis.

[59] Yang T. (2019). Preliminary Study on the Ecological Cause and Distribution Pattern of Species Diversity in *Lysimachia* (*Primulaceae*). Master’s Thesis.

[60] Tang Q., Li M., Xiao S., Li F., Hu S., Liu Y., Shan Z., Xu Y., Zhou Y. (2025). Geographical distribution and potential distribution area prediction of *Epimedium koreanum* Nakai. and *Epimedium brevicornu* Maxim. in China. World Chin. Med..

[61] Mi W., Ren W., Chen Z., Zhang M., Liu Y. (2023). Prediction of geographical distribution of three thyme species in China under future climate scenarios. Chin. J. Grassl..

[62] Peng D., Yang L.E., Yang J., Li Z. (2021). Seed dormancy and soil seed bank of the two alpine *Primula* species in the Hengduan Mountains of southwest China. Front. Plant Sci..

[63] Caser M., Lovisolo C., Scariot V. (2017). The influence of water stress on growth, ecophysiology and ornamental quality of potted *Primula vulgaris* ‘Heidy’ plants. New insights to increase water use efficiency in plant production. Plant Growth Regul..

[64] Yang B. (2015). Species Richness Distribution Patterns of *Primula* L. and Its Affecting Factors. Master’s Thesis.

[65] Kong L., Huang M., Liu C., Wang S., Liu D., Wang R. (2025). Analysis of suitable habitat evolution for northern Magnoliaceae plants under climate change scenarios. Acta Ecol. Sin..

[66] Nawkar G.M., Maibam P., Park J.H., Sahi V.P., Lee S.Y., Kang C.H. (2013). UV-induced cell death in plants. Int. J. Mol. Sci..

[67] Sakalauskaite J., Viskelis P., Dambrauskiene E., Sakalauskiene S., Samuoliene G., Brazaityte A., Duchovskis P., Urbonaviciene D. (2013). The effects of different UV-B radiation intensities on morphological and biochemical characteristics in *Ocimum basilicum* L.. J. Sci. Food Agric..

[68] Paul N.D., Moore J.P., McPherson M., Lambourne C., Croft P., Heaton J.C., Wargent J.J. (2012). Ecological responses to UV radiation: Interactions between the biological effects of UV on plants and on associated organisms. Physiol. Plant.

[69] Meyer P., Van de Poel B., De Coninck B. (2021). UV-B light and its application potential to reduce disease and pest incidence in crops. Hortic. Res..

[70] Yao X.Q., Chu J.Z., He X.L., Si C. (2014). The effects of UV-B radiation intensity on biochemical parameters and active ingredients in flowers of Qi Chrysanthemum and Huai Chrysanthemum. Photochem. Photobiol..

[71] Wu Q., Dong S.B., Yang L., Qi X.J., Zhang Y., Yang M.Q., Cheng J. (2024). Prediction of potential distribution of *Cypripedium macranthos* under climate change scenarios in China. Acta Ecol. Sin..

[72] Xie T., Shi H., Guo Q., Wang T., Zou Q., Wei M., Liu C., Huang J., Su Y., Yang C. (2010). A medicinal plant highly stable for survival under climate change due to UV buffering—*Chrysanthemum indicum* L. future adaptation analysis. Flora.

[73] Liang Q., Xu X., Mao K., Wang M., Wang K., Xi Z., Liu J. (2018). Shifts in plant distributions in response to climate warming in a biodiversity hotspot, the Hengduan Mountains. J. Biogeogr..

[74] Xie H., Burgess K.S., Yang X.-F., Ahrends A., Gao L.-M., Li D.-Z. (2019). Distributional responses to climate change for alpine species of *Cyananthus* and *Primula* endemic to the Himalaya-Hengduan Mountains. Plant Divers..

[75] Xia M., Chi X., Yu J., Han Y., Han S., Chen S., Li Y., Zhang F. (2025). Genetic structure and conservation implications of *Lancea tibetica* (*Mazaceae*), a traditional Tibetan medicinal plant endemic to the Qinghai-Tibet Plateau. BMC Plant Biol..

[76] He X., Burgess K.S., Yang X.-F., Ahrends A., Gao L.-M., Li D.-Z. (2019). Upward elevation and northwest range shifts for alpine *Meconopsis* species in the Himalaya-Hengduan Mountains region. Ecol. Evol..

[77] Kumari P., Wani I.A., Khan S., Verma S., Mushtaq S., Gulnaz A., Paray B.A. (2022). Modeling of *Valeriana wallichii* habitat suitability and niche dynamics in the Himalayan region under anticipated climate change. Biology.

[78] Gavin D.G., Fitzpatrick M.C., Gugger P.F., Heath K.D., Rodríguez-Sánchez F., Dobrowski S.Z., Hampe A., Hu F.S., Ashcroft M.B., Bartlein P.J. (2014). Climate refugia: Joint inference from fossil records, species distribution models and phylogeography. New Phytol..

[79] Xie X., Yan H., Wang F., Ge X., Hu C., Hao G. (2012). Chloroplast DNA phylogeography of *Primula ovalifolia* in central and adjacent southwestern China: Past gradual expansion and geographical isolation. J. Syst. Evol..

[80] Wang F.Y. (2007). Phylogeography of Representative Species of *Primula* in the Eastern Himalaya-Hengduan Mountains Region. Master’s Thesis.

[81] Shi H.H. (2022). Global Geographical Patterns and Causes of Species Richness and Breeding Systems in *Primula*. Master’s Thesis.

[82] Yan H.F., Wang F.Y., Hao G. (2009). Progress in conservation genetics and phylogeography of *Primula*. Guihaia.

[83] Lengyel S., Gove A.D., Latimer A.M., Majer J.D., Dunn R.R. (2010). Convergent evolution of seed dispersal by ants, and phylogeny and biogeography in flowering plants: A global survey. Perspect. Plant Ecol. Evol. Syst..

[84] Song M., Yue S., Sun H., Li Z. (2011). Phylogeography of *Primula poissonii* in the Hengduan Mountains region. Plant Divers. Resour..

[85] Huan Z.Q., Geng X.M., Xu X.R., Liu W., Zhu Z.L., Tang M. (2023). Potential geographical distribution of *Michelia martinii* under different climate change scenarios. J. Ecol. Rural Environ..

[86] Richards A.J. (2003). Primula.

[87] Zhao W.F., Zong L.P., Wang M.J. (2024). Spatial distribution of nature reserves in China. Acta Ecol. Sin..

[88] Zhang Y., Tang J., Ren G., Zhao K., Wang X. (2021). Global potential distribution prediction of *Xanthium italicum* based on Maxent model. Sci. Rep..

[89] Xu Y., Zang R.G. (2022). Advances in Conservation Theory and Practice of Plant Species with Extremely Small Populations in China. Biodivers. Sci..

[90] Yang W., Li Y., Zhang S., Yu C., Kang H., Shi F., Chen Y., Zhang K. (2016). The Construction Practice of the First Protection Community for Wild Plant Species with Extremely Small Populations in China: The Yunnan *Nyssa yunnanensis* Protection Community. J. West China For. Sci..

[91] Boria R.A., Olson L.E., Goodman S.M., Anderson R.P. (2014). Spatial filtering to reduce sampling bias can improve the performance of ecological niche models. Ecol. Model..

[92] Sang Y., Ren H.L., Shi X., Xu X., Chen H. (2021). Improvement of Soil Moisture Simulation in Eurasia by the Beijing Climate Center Climate System Model from CMIP5 to CMIP6. Adv. Atmos. Sci..

[93] Tan J., Huang A., Shi X., Zhang Y., Zhang Y., Cao L., Wu Y. (2022). Evaluating the Performance of BCC-CSM2-MR Model in Simulating the Land Surface Processes in China. Plateau Meteorol..

[94] Zhang X., Xue F., Dong X., Lin R. (2022). Subseasonal Evolution of the East Asian Summer Monsoon Simulated by Two BCC Climate Models Participating in CMIP6. Clim. Environ. Res..

[95] Li N.Q., Xu G.Y. (2020). Grid analysis of land use based on natural breaks (jenks) classification. Bull. Surv. Mapp..

[96] Yan X., Wang S., Duan Y., Han J., Huang D., Zhou J. (2021). Current and future distribution of the deciduous shrub *Hydrangea macrophylla* in China estimated by MaxEnt. Ecol. Evol..

[97] Zhao R., Wang S., Chen S. (2024). Predicting the potential habitat suitability of *Saussurea* species in China under future climate scenarios using the optimized MaxEnt model. J. Clean. Prod..

[98] Guo Y., Ren L., Zhang S., Tian X., Tang S., Sun Z., Pan J., Zhang Z. (2023). Analysis of the prediction of the suitable distribution of *Polygonatum kingianum* under different climatic conditions based on the MaxEnt model. Front. Earth Sci..

[99] Yang G., Liu N., Zhang X., Zhou H., Hou Y., Wu P., Zhang X. (2024). Prediction of the potential distribution of *Chimonobambusa utilis* (*Poaceae*, *Bambusoideae*) in China, based on the MaxEnt model. Biodivers. Data J..

[100] Huang D., An Q., Huang S., Tan G., Quan H., Chen Y., Zhou J., Liao H. (2023). Biomod2 modeling for predicting the potential ecological distribution of three *Fritillaria* species under climate change. Sci. Rep..

[101] Shi X., Wang J., Zhang L., Chen S., Zhao A., Ning X., Fan G., Wu N., Zhang L., Wang Z. (2023). Prediction of the potentially suitable areas of *Litsea cubeba* in China based on future climate change using the optimized MaxEnt model. Ecol. Indic..

[102] Kang Y., Lin F., Yin J., Han Y., Zhu M., Guo Y., Tang F., Li Y. (2025). Projected distribution patterns of *Alpinia officinarum* in China under future climate scenarios: Insights from optimized Maxent and Biomod2 models. Front. Plant Sci..

[103] Li L., Wu H., Gao Y., Zhang S. (2023). Predicting ecologically suitable areas of cotton cultivation using the MaxEnt model in Xinjiang, China. Ecologies.

